# Including significant others in vocational rehabilitation: a scoping review

**DOI:** 10.3389/fresc.2026.1833648

**Published:** 2026-06-22

**Authors:** Franziska Weißenstein, Eileen Wengemuth, Larissa Fink, Lukas Kühn, Kyung-Eun (Anna) Choi

**Affiliations:** 1 Center for Health Services Research, Brandenburg Medical School Theodor Fontane, Neuruppin, Germany; 2 Department of Therapeutic Health Professions, University Hospital Münster, Münster, Germany; 3 Health Services Research, Research Center Medical Imaging and Artificial Intelligence (MIAAI), Danube Private University (DPU) GmbH, Krems-Stein, Austria

**Keywords:** family caregivers, family interventions, return to work, significant others, social support, vocational rehabilitation

## Abstract

**Purpose:**

Significant others (SOs) play an important role in health, recovery processes and in the return to (or stay at) work. However, the extent to which they are included in rehabilitation programs, especially those aimed at enabling participation in working life, is unclear. This scoping review's objective is to create an overview of research on vocational rehabilitation interventions including SOs and to assess in what way SOs are addressed in these.

**Methods:**

This scoping review was guided by the framework of Arksey and O’Malley. A two-stage search was conducted in Medline (PubMed), Web of Science, PsycINFO, and Social Sciences Citation Index, including studies which addressed vocational rehabilitation approaches involving SOs. Data was extracted using a charting table based on the TIDieR-Rehab checklist, capturing study characteristics, intervention details, characteristics of SOs and the conceptual basis for including SOs.

**Results:**

Eight studies published between 1985 and 2024 were included. They adopted different quantitative, qualitative and mixed-methods approaches. One group of interventions directly targeted families of rehabilitants while a second group focused on rehabilitation professionals. A third group investigated the conceptual or contextual background. The interventions varied regarding what kind of rehabilitants they were tailored for. Most did not conceptualize their rationale for including SOs.

**Conclusion:**

This scoping review shows that the inclusion of SOs in vocational rehabilitation remains rare. Existing interventions are heterogeneous and rarely concept-driven. Future research should focus on the development and evaluation of structured, theory-informed approaches that integrate SOs in a targeted and needs-based manner, while also considering potential burdens and conflicting roles.

## 1 Introduction

Significant others (SOs) are individuals who may influence another person, particularly through socialization processes that shape social skills, beliefs, values, and other behaviours (1). SOs - such as relatives, partners, or close friends - play a key role in health, recovery processes, and adjustment to chronic conditions, and in maintaining or regaining work participation (2). The importance of social relationships is widely recognized in clinical and rehabilitation settings, and several diagnostic systems include measures of social support to assess individuals’ resources and vulnerabilities in coping with health challenges (3).

Social support can be understood as a higher-order construct comprising both structural and functional aspects. Structural dimensions describe the quantitative features of a social network - such as size, density, and contact frequency and include related concepts such as social integration, social relationships, social embeddedness, and network connectedness (4, 5).

Functional components, in contrast, refer to the different types of supportive behaviours. These typically include emotional, informational, and instrumental support - each influencing health outcomes in distinct ways (6). Functional support can also be conceptualized in terms of received support (actual supportive behaviours experienced in the past), perceived availability of support (subjective expectations about future support), and satisfaction with support (evaluations of how beneficial the support actually is) (7, 8).

An increasing number of studies report an association between supportive social networks and key rehabilitation outcomes. These include adjustment to disability, overall well-being, mental health, vocational success, adherence to medical treatment, and survival rates among individuals with disabilities or chronic health conditions (8, 9).

Nonetheless, in the context of chronic illness, social support should not be viewed solely as a beneficial coping resource. From the rehabilitants’ perspective, dependence on help in daily activities may be experienced as a loss of autonomy. It can also involve difficulty in meeting the expectations of SOs and uncertainy about appropriate behaviour within social networks (10). Some individuals also experience increasing conflicts with friends or partners due to unwanted social support (11). In a qualitative study by Palant and Himmel (12), patients with chronic diseases reported negative experiences with social support, including receiving unwanted illness information and distressing comparisons with other patients. Participants expressed frustration about not being treated normally, feeling pitied or stigmatized, and perceiving their social networks as either overreacting to or being indifferent toward their condition.

At the opposite end of the spectrum, illness can sometimes bring unconscious benefits, such as receiving more attention from others, being relieved of certain daily obligations or responsibilities, and receiving increased support. Such benefits may discourage patients from fully engaging in their treatment and recovery (13).

These ambivalent effects of social support are particularly relevant for work participation among individuals with chronic conditions. A systematic review by Snippen et al. (9) found that SOs’ attitudes and behaviours can both facilitate and hinder work participation among employees with chronic conditions. Positive, supportive attitudes and open communication promote job retention and a successful return to work. In contrast, negative perceptions and overprotective behaviours may impair employment and work functioning. These findings highlight the importance of systematically involving SOs in the rehabilitation. They should be seen as potential resources, but also as possible barriers to recovery or return-to-work processes when appropriate.

Although evidence suggests that clinical interventions involving SOs are more effective than standard care (14–16), few studies have examined such approaches in vocational rehabilitation settings. This review aims to provide an overview of research on vocational rehabilitation interventions that include SOs. It also examines how SOs are conceptualized within these interventions - as resources, barriers, sources of social support, or as individuals affected by the rehabilitation process. This exploratory approach helps identify the range of intervention designs and highlights gaps in current research.

Three main objectives guided this review: (1) to identify and examine how vocational rehabilitation interventions are designed to include rehabilitants’ SOs, (2) to describe the target populations of rehabilitants and SOs within these interventions, and (3) to describe how their roles are conceptualized across different intervention approaches.

## 2 Method

This review was conducted by a multidisciplinary team with established expertise in health services research, rehabilitation sciences, and psychology. The scoping review followed the five-stage methodological framework developed by Arksey and O'Malley (17): (I) identifying the research question(s), (II) identifying relevant studies, (III) selecting studies based on predefined inclusion and exclusion criteria, (IV) charting the data, and (V) collating, summarizing, and reporting the results. The review was conducted and reported in accordance with the PRISMA Extension for Scoping Reviews (PRISMA-ScR) checklist (18) which is provided in Supplementary Table 1. To ensure methodological transparency, the review protocol was preregistered on the Open Science Framework (https://doi.org/10.17605/OSF.IO/46P2E).

### 2.1 Eligibility criteria

To be included in the review, studies or protocols had to report on vocational rehabilitation approaches that involved SOs, meaning any form of effort to include rehabilitants’ SOs, such as spouses, partners, family members, other relatives, friends, or neighbors, in rehabilitation aimed at participation in working life. While we included some vocational rehabilitation accessing groups, such as students transitioning from school to work, we only sought literature that explored vocational rehabilitation use and did not restrict our search to any specific groups, e.g., based on a specific condition. Studies reported in English or German were included. Furthermore, studies were eligible if they explored the views and experiences of rehabilitants, their SO's, or rehabilitation professionals on the inclusion of SOs in vocational rehabilitation processes. No restrictions were placed on the type of health condition, disability, or specific work transition phase, intervention delivered, country, study design, or year of publication, as the review aimed to capture the full range of vocational rehabilitation interventions involving SOs. Studies were excluded if they were reported in languages other than English or German, or if they addressed the inclusion of SOs in rehabilitation interventions not specifically aimed at participation in working life. The analysis excluded grey literature, since it is typically published at the national level without any available English translation. Studies were also excluded if they reported only general findings on the relevance or influence of SOs or social support for return to work without specifically focusing on vocational rehabilitation programs or interventions. Opinion papers and editorials were excluded from the review as they do not provide detailed information of vocational rehabilitation interventions or programs that involve SOs.

### 2.2 Search strategy

**Figure 1:**
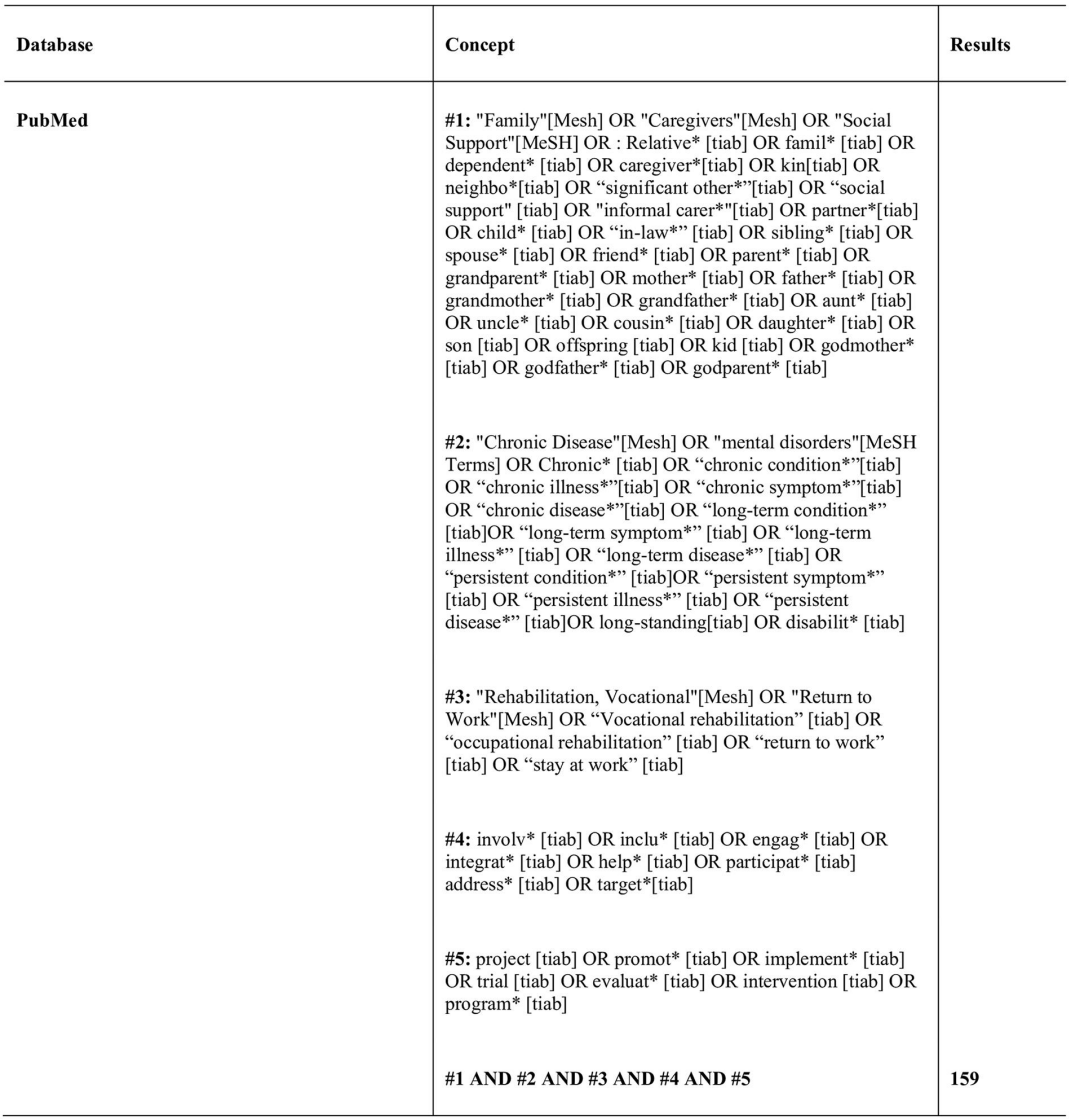
Representative search terms used in pubMed.

Eligible studies were systematically identified through a literature search using the following databases for English and German language studies research: Medline (PubMed), Web of Science, PsycInfo and the Social Sciences Citation Index (via Web of Science). The search terms included keywords and index terms derived from the key concepts of this review. Through iterative testing and refinement of search strategies, four main components were identified as most effective for retrieving relevant studies: SOs, chronic conditions, vocational rehabilitation, involvement and interventions. Synonyms related to these components were combined to maximize the capture of relevant literature. Searches were adapted for each database to accommodate differences in search syntax and controlled vocabulary. A representative example of the search string used for PubMed is provided in Figure 1. The complete search strategy for all databases is provided in Supplementary Table 2.

Keywords and index terms within each domain were combined using the Boolean operator ‘OR’. Where appropriate, truncation was used. To connect the different domains, the Boolean operator ‘AND’ was used.

The screening of retrieved articles was conducted in two stages: In the first stage, two reviewers (EW, FW) independently screened the titles and abstracts against the defined eligibility criteria. Any disagreements were resolved through discussion, and there was no need for a third reviewer as the reviewers reached a mutual agreement. In the second stage, a full-text screening was performed, including a third reviewer (LF) following the same process as the first stage, to determine the final set of included articles. All Data were managed using the online tool RAYYAN Version 2021 (Cambridge, USA).

### 2.3 Data extraction and charting

A data charting table was developed based on the TIDieR-Rehab checklist (19), with additional categories developed by the research team (EW, FW, and LF). The table was pilot-tested using three randomly selected articles from the included studies to ensure its applicability and consistency. Adaptations to the charting table were discussed by consensus between the reviewers. The final charting tool comprised four main categories, each organized in a dedicated table. The first table captured essential publication details such as author, year, country) as well as study details including study design, intervention type, study aim, sample, outcome measures, quantitative and qualitative results, conclusions, and methodological limitations. The second table on intervention characteristics was based on and adapted from the TIDieR-Rehab Checklist and focused on rationale/theory and intervention target, content (materials and procedures) and delivery parameters (provider, mode, setting, timing, duration). The third table described the participant characteristics of the study and approaches to SO involvement: rehabilitant characteristics, SO selection and recruitment approaches, and the integration of SOs (targeted components, assigned roles, initiator, and timing of involvement).

Based on this charting table, results were further synthesized and narratively illustrated in additional tables and figures of which each aimed at addressing one of the stated objectives. Since scoping reviews are designed to provide an overview of existing evidence without assessing the methodological quality, the research team chose not to perform a formal appraisal of the included studies. However, regarding research question three, a table was developed to assess the conceptualization of involvement of SOs. This table was conceptualized to provide a quick overview of how SOs are addressed across the included studies. It systematically captures whether a theoretical basis for including SOs is specifically mentioned, whether SOs are explicitly targeted by interventions, whether training or guidance is offered to SOs, and how their roles are defined.

## 3 Results

### 3.1 Literature search

**Figure 2:**
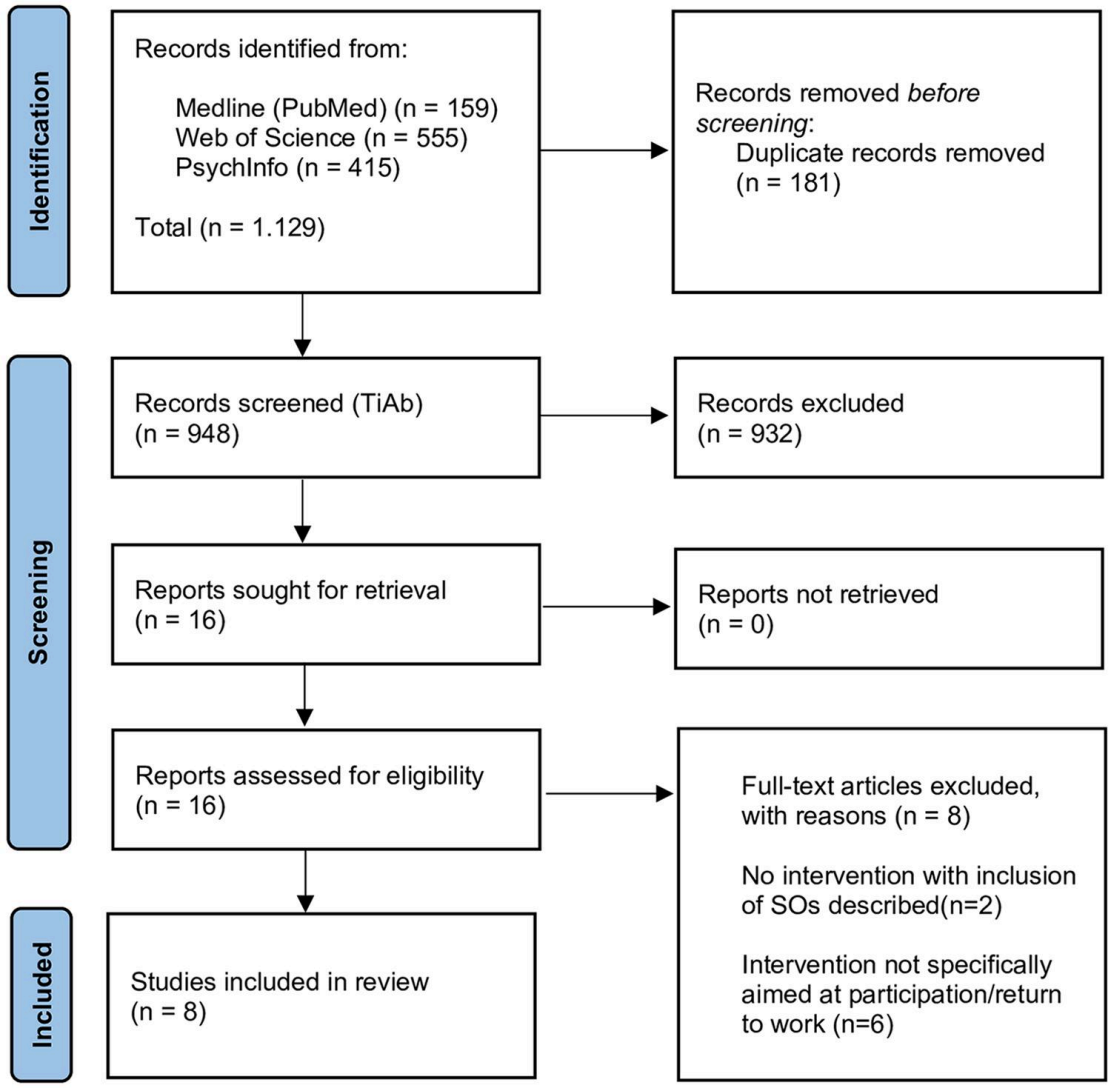
Flow chart illustrating the study selection process.

We conducted a search using the above-mentioned search terms on the electronic databases Medline (PubMed), Web of Science and PsycInfo on 14th of May 2025 and identified 1.129 records in total. After duplicate removal, we screened the titles and abstracts of 948 records. Out of these, we assessed 16 full texts for eligibility. A final set of eight articles met the exclusion and inclusion criteria of the literature search. The study selection process is illustrated in Figure 2.

### 3.2 Characteristics of included studies

The included studies were published between 1985 and 2024, with the majority originating from the USA (20–23), three from the Netherlands (2, 24, 25), and one from Denmark (26).

Various methodological approaches were applied to investigate the involvement of SOs in vocational rehabilitation at different levels. Two studies used qualitative methods, specifically focus groups, to explore perspectives on SO involvement in return-to-work processes, one examining providers’ perspectives and the other exploring workers’ perspectives with chronic conditions (2, 20). The efficacy of two interventions was evaluated or planned to be evaluated using randomized controlled trial designs (25, 26). One study used a mixed-methods feasibility design to assess the practicability of family group conferences (24). Additionally, a descriptive case study outlined a step-by-step procedure for transitioning from school to work (22). Another non-empirical study presented a conceptual paper outlining a three-stage intervention model based on the authors’ professional experience, illustrated by a case study (23). Furthermore, one review synthesized research on family support in rehabilitation conducted between 1980 and 1990, incorporating findings from observational studies, non-randomized and randomized intervention studies, case studies and surveys (21). An abbreviated version of the extraction sheet regarding the study characteristics can be found in Table 1; the full extraction sheets can be found in the Supplementary Table 3.

**Table 1:** Study characteristics.

Publication details	Study details	Authors’ conclusions
Reference: Snippen et al. (2023) (25) Title: Training for occupational health physicians to involve significant others in the return-to-work process of workers with chronic diseases: a randomized controlled trial Country: Netherlands	Study design: non-blinded randomized controlled trial (RCT), with an intervention and a wait-listed control group. Aim of the study: evaluate the effectiveness of an e-learning training module for occupational health physicians regarding involving significant others in the return-to-work process of workers with chronic diseases	Improved physicians’ knowledge & motivation, self-efficacy concerning the involvement of SOs. Practical tips and materials were valued. Criticism: navigation issues, tech problems, lack of practice, feedback, and peer exchange.
Reference: Brongers et al. (2020) (24) Title: Feasibility of Family Group Conference to promote return-to-work of persons receiving work disability benefit Country: Netherlands	Study Design: Mixed-methods feasibility pilot study (no control group) with measurements at baseline (T0), immediately post-intervention (T1), 3 months (T2), and 6 months (T3) follow-up Aim of the Study: evaluate the feasibility of Family Group Conference	Family Group Conference facilitated return to work (RTW) planning; positive experiences reported, perceived as helpful by majority.
Reference: Hoeffding et al., (2017) (26) Title: A manual-based vocational rehabilitation program for patients with an acquired brain injury: study protocol of a pragmatic randomized controlled trial (RCT) Country: Denmark	Study Design: protocol for an interventional, two-arm, six-month follow-up, cluster randomized controlled trial. Control group: conventional vocational rehabilitation (VR) Aim of the Study: develop an individually targeted manual-based VR program and determine its efficacy for patients with an acquired brain injury (ABI)	none yet
Reference: Chang et al., (2024) (20) Title: Providers’ Perceptions of the Collaborative Challenges and Assistance Provided to Families in Pre-Employment Transition Services Country: USA	Study Design: qualitative, focus groups Aim of the Study: explore providers’ perspective on family involvement in pre-employment transition planning	Providers emphasize tailored direct support for families, culturally responsive communication, and community resource development to enhance Pre-Employment Transition Services family collaboration.
Reference: McKenna & Power (2000) (23) Title: Engaging the African American Family in the Rehabilitation Process: An Intervention Model for Rehabilitation Counselors Country: USA	Study Design: not a study *per se*, sharing long-term professional experiences Aim of the Study: developing an intervention model from long-term experience	Rehabilitation counselors should have cultural awareness and need to adopt different roles in the process.
Reference: McCarthy et al., 1985 (22) Title: Transition from School to Work: Developing the Process for Individuals with Severe Disabilities Country: USA	Study Design: step by step procedure to develop a transition process, descriptive case study Aim of the Study: developing model for school-to-work transition process	Employment for individuals with severe disabilities is achievable if transition planning is individualized, starts early, and involves parents, schools, and community agencies.
Reference: Snippen et al., 2022 (2) Title: Workers’ views on involving significant others in occupational health care: a focus group study among workers with a chronic disease Country: The Netherlands	Study Design: Qualitative focus group study Aim of the Study: To explore workers’ views on involving significant others in occupational health care in the context of chronic disease	Involving SOs can be valuable in occupational health care for workers with chronic disease, but requires careful consideration of privacy, voluntariness, and clear role definition.
Reference: Kelley & Lambert, 1992 (21) Title: Family Support in Rehabilitation: A Review of Research, 1980–1990 Country: USA	Study Design: Narrative review: includes observational, nonrandomized & randomized intervention studies, case studies & surveys. Aim of the Study: to review and synthesize research on the role of family support in rehabilitation for persons with chronic illnesses and disabilities, published between 1980 and 1990.	Family support is critical for rehabilitation outcomes; Positive effects when families provide supportive involvement; risks when families are overprotective or dysfunctional. Emphasizes structured family involvement as best practice; systematic, controlled studies with standardized measures needed.

### 3.3 Characteristics of rehabilitants and their SOs

While all interventions aim to include the SOs into the vocational rehabilitation process, they differ regarding what kind of rehabilitants they are focussing on, who exactly is envisioned as possible SOs as well as how their roles are conceptualized.

Two interventions focused on students with disabilities reaching the end of their high school education (20, 22). Three targeted persons of working age with various chronic diseases in different employment statuses (sick leave/ unemployed/ employed) (2, 24, 25). Other interventions took a diagnosis-specific approach and included patients with an acquired brain injury (26) or focused on a specific ethnic group (23). The included review (21) reported on rehabilitants from varied employment statuses, ages and diagnoses.

Most interventions do not have strict selection criteria or for who counts and can be included as a SO nor an official recruitment process. Many emphasized that the SO involvement should not be imposed by the professionals. Mostly, family members were thought to be the relevant SOs. In the case of students, their parents or guardians were addressed. In most cases, the rehabilitants selected the SO to be included themselves. However, one intervention targeted the parents directly (22). Only one intervention made the willingness of at least two SOs, among them one family caregiver, to participate, mandatory (26).

The studies also vary in how they specify the exact role assigned to the SOs in the interventions they describe (and thus also the motivation for including them at all). In most of the interventions, SOs are included because they are considered to be a potential resource, can provide support for the rehabilitant and thus aid the return to (or stay at) work process. Some also see the SO as a potential mediator between health professionals and rehabilitants, who improves the communication, provides information, fosters the anchoring of the rehabilitation and acts as a resource for professionals. Chang et al. (20) point out that for the case of students with disabilities including the parents is legally mandatory. Kelley & Lambert (21) also name involving SOs as a strategy to prevent dysfunctional family patterns from being a potential barrier to the rehabilitants’ return to work. One study (26) also mentions the aim of reducing the caregiver burden.

The intervention components who directly target SOs range from education, training or counselling to jointly devising return-to-work plans. An overview of rehabilitant and SO characteristics and roles can be found in Table 2.

**Table 2:** Characteristics of rehabilitants and SOs.

Reference	Characteristics of rehabilitants	Selection criteria and recruitment for SOs	Assigned roles of SOs	Components of the intervention targeting/ including SOs
Snippen et al. (25)	Patients in the RTW Process. Unemployed/ on sick leave.	worker-initiated and consent-based	Emotional support, information provision, collaborative planning.	No formal psychoeducation or skills training mentioned for SOs.
Brongers et al. (24)	Unemployed, receiving work disability benefit.	Clients selected members of their social network (family, friends) for the Family Group Conference.	In Family Group Conference, to help with RTW Process.	Development of a RTW Plan during Family Group Conference.
Hoeffding et al. (26)	Patients with acquired brain injury.	Minimum 2 family members for family intervention + 1 primary family caregiver for active participation in individual sessions.	Positive effect on the reintegration, valuable resource for professional support & ensure future consolidation and anchoring of the VR. Reduce caregiver burden.	Manualized family intervention program & individual caregiver coaching. Supporting the caregiver in assisting the patient
Chang et al. (20)	Students with disabilities at the transition from high school to postsecondary education/ work.	Parents	Legally parents of youth with disabilities need to be included. Counted on as a resource.	counseling
McKenna & Power (23)	African Americans with disabilities.	Families of person with disability	support/ resource	joint goal setting
McCarthy et al. (22)	Special education students with disabilities preparing for employment. Age: 16–22 years.	Parents/guardians recruited via student or teachers for transition team.	part of transition team supporting students as support/ resource	Parent training/education programs Joint goal-setting in transition team and Individual Transition Plan meetings.
Snippen et al. (2)	Workers with various chronic diseases. Of working age. Male and female.	No formal selection, recruitment by worker's choice, not imposed by professionals. OHPs inform workers about the option and potential benefits.	Resource: support, encouragement, practical help Mediator: improve communication between worker and OHP	Present in sessions with OHP
Kelley & Lambert (21)	Different employment statuses. Ages ranging from children to elderly. Diagnoses: Physical disabilities, long-term mental illnesses, substance abuse, developmental disabilities.	Primarily via patient identification/choice. No formal criteria. Voluntary involvement emphasized, not imposed by professionals.	Resource functions: emotional support, daily care assistance, collaborative planning, communication facilitation Barrier: dysfunctional family patterns (overprotection, enmeshment)	Psychoeducation, Skills training, multifamily groups, skill-sharing, family counseling. Information: pre-discharge education on coping and prevention strategies.

### 3.4 Intervention characteristics

The included studies illustrate that family involvement in vocational rehabilitation is approached in diverse ways. Rather than representing variations of a single intervention type, the studies reflect distinct understandings of how families or significant others relate to the vocational rehabilitation process. Across interventions, differences emerge in the extent to which families are directly engaged, supported through professional mediation, or addressed at a broader conceptual or contextual level. The following sections group the interventions according to these underlying approaches to family involvement.

#### 3.4.1 Interventions directly involving families or SOs

Two studies implemented vocational rehabilitation approaches in which SOs were formally included in the intervention process. The *Family Group Conference* model (24) brings together clients receiving work disability benefits and selected members of their social network to jointly develop a return-to-work plan. The procedure includes preparation, a facilitated meeting, a period of “private family time,” and structured follow-up. Similarly, the manual-based vocational rehabilitation program for individuals with acquired brain injury (26) integrates an eight-session family intervention and individual caregiver coaching. Both approaches involve families as active participants and resources in planning or supporting vocational rehabilitation, though neither includes formal training materials specifically for SOs.

#### 3.4.2 Professional-focused interventions supporting the inclusion of SOs

A second group consists of interventions that aim to strengthen professionals’ competencies to SOs rather than them directly. The TOTIS e-learning module (25) provides occupational health physicians with structured content, materials, and guidance on when and how to address the role of SOs in return-to-work processes. In addition, McKenna and Power (23) propose a counseling-based model for rehabilitation professionals working with African American families, emphasizing culturally responsive family engagement. In both cases, the primary targets are practitioners; SOs are indirectly affected through improved professional practice.

#### 3.4.3 Contextual and conceptual approaches to family involvement in vocational rehabilitation

A third group of studies does not present a specific vocational rehabilitation intervention, but instead focuses on the broader context in which the involvement of SOs takes place. Chang et al. (20) examine how providers of Pre-Employment Transition Services perceive barriers and facilitators to engaging families of youth with disabilities. While Snippen et al. (2) directly explore the perspectives and preferences of workers with chronic diseases regarding the involvement of significant others in occupational health care. McCarthy et al. (22) outline a coordinated transition model involving schools, families, and rehabilitation services to support the move from school to work. Finally, the narrative review by Kelley and Lambert (21) synthesizes various family-oriented rehabilitation approaches and highlights common elements such as psychoeducation, communication training, and problem-solving support. These studies do not describe stand-alone interventions but provide insight into structural, theoretical, or system-level approaches relevant to the involvement of SOs.

Across all groups, the interventions differ in their degree of direct SO involvement, the extent to which materials or guidance are provided, and whether the primary target is the rehabilitant, the family, or the professional team. An overview of the intervention characteristics can be found in Table 3; the full extraction sheets can be found in the Supplementary Table 3.

**Table 3:** Intervention characteristics.

Reference	Intervention target	Procedures	Delivery parameters
Snippen et al. (25)	Occupational health physicians	Education on when and how to address the role of significant others, coping and re-integration, the role of dyadic coping, the role of illness perceptions	Timing: is left to the trained OHP's clinical judgment Who provided: researchers Where: online module
Brongers et al. (24)	participants aged 17–65 years, receiving work disability, benefit, had (partial) capacity to reintegrate into paid work	Education/ information provision, discussion of and agreement on a return-to-work plan, monitoring of plan execution	Timing: not linked to the onset of the condition Who provided: Labour Experts from the Social Security Institute Family Group Conference facilitator Where: Community setting, typically in participants’ home or chosen venue
Hoeffding et al. (26)	patients with acquired brain injury and their family caregivers	family intervention program, individual caregiver coaching	Timing: 3–24 months after acquiring the brain injury Who provided: rehabilitation team (profession not specified) Where: municipality/ workplace/ home
Chang et al. (20)	youth with disabilities before transitioning into work or post-secondary education and their families	counselling	Timing: transition period from high school to postsecondary education or work Who provided: VR counselors, transition educators Where: unclear, probably schools, homes or VR institutions
McKenna & Power (23)	Rehabilitation professionals counselling black persons with disabilities and their families	counselling, assessment, provision of information	Timing: not specified Who provided: rehabilitation counselors Where: unclear, probably schools, homes or VR institutions
McCarthy et al. (22)	youth with disabilities/ mental retardation who are transitioning from school programs to employment.	assessment, counseling, education	Timing: age 16, during secondary school Who provided: special education teachers, Rehabilitation counselors/case managers, disability support services Where: schools/ job site, agencies, and family settings
Snippen et al. (2)	Workers with chronic illness and their significant others (partners, family, close friends).	guided integration of SO into occupational health care systems by professionals	Timing: not specified: Potentially at various stages of occupational health care: diagnosis, return-to-work planning, ongoing work support. Who provided: occupational health professionals Where: occupational health care
Kelley & Lambert (21)	Persons with chronic illness/disability (physical, mental, substance abuse, developmental) and their significant family members	Physical illnesses: pre-discharge education for caregivers; Mental illness: Inpatient Family Intervention, psychoeducation, communication/problem-solving, medication compliance; Substance abuse: counseling, social support; Developmental: case management, coping skills, behavioral parent training	Timing: Pre-discharge from hospital/inpatient settings/ during acute rehabilitation/ post-hospital/community transition/ throughout rehab for chronic conditions. Early intervention for spinal cord injuries counseling. General: flexible, across the rehabilitation continuum. Who provided: Rehabilitation professionals (social workers, physicians, therapists, psychologists, psychiatrists, nurses, case managers, sometimes volunteers) Where: Hospitals, inpatient rehab units, outpatient clinics, patient homes, community centers

### 3.5 Conceptualization of the involvement of SOs

To get a quick overview, Table 4 was created to systematically compare the extent to which the inclusion of SOs is theoretically grounded, explicitly targeted, and actively supported across the included studies. This underlines the diversity in conceptualisation and operationalisation of SO involvement in vocational rehabilitation or return-to-work interventions.

**Table 4:** SO involvement: theory, targeting, and training.

SO involvement: theory, targeting, and training	Snippen et al. (25)	McCarthy et al. (22)	Brongers et al. (24)	Hoeffding et al. (26)	Chang et al. (20)	McKenna and Power (23)	Snippen et al. (2)	Kelley and Lambert (21)
Is there a theoretical basis or rationale for including SOs?	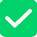	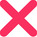	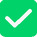	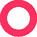	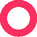	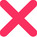	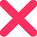	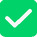
Are SOs explicitly targeted in the intervention described in the study?	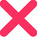	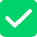	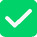	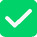	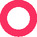	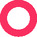	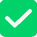	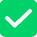
Are SOs given training, materials, guidance?	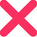	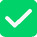	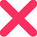	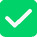	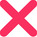	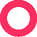	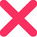	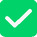

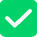
, Yes; 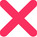, NO; 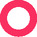, unclear/not specified.

As mentioned above, since the included studies use different approaches to incorporate SO, they also vary in how they conceptualize the involvement of SO. This ranges from explicit theoretical models to retrospective theories, or even none at all.

Some studies, such as the e-learning tool for occupational health physicians by Snippen et al. (25), are incorporating concepts like “illness perceptions” and “dyadic coping” (27) theory helping physicians to understand and support the role of SOs in recovery and return-to-work processes. Brongers et al. (24) Family Group Conference intervention explicitly links their Family Group Conference intervention to theoretical frameworks such as empowerment theory, strength-based approaches, and social network theory (28). In contrast, McCarthy et al. (22), McKenna & Power (23), Snippen et al. (2) and the study protocol by Hoeffding et al. (26) do not present a theoretical basis, while Chang et al. (20) are referencing a theoretical concept in retrospect using Vygotsky & Cole (29) zone of proximal development to explain how support can help families move from what they can do alone to what they can achieve with assistance. In practice, this means matching support to families’ goals and providing help that genuinely strengthens their abilities (30). Kelley & Lambert (21) found that there were few theoretical frameworks or concepts supporting the SOs’ interventions. The stress-buffering model was one of the most frequently referenced concepts in the included studies, but primarily as a *post-hoc* explanation rather than to guide intervention design. Other psychiatric rehabilitation studies reference the vulnerability–stress model, which conceptualises family behaviour as a stressor that interacts with patient vulnerability. However, the authors conclude that the variety of family support types and sources makes it difficult to develop a coherent theory of how social support is most effective.

The reviewed studies demonstrate significant variation in the extent to which SOs were explicitly targeted and the level of support provided to them. McCarthy et al. (22) directly involve SOs by offering parental training that addresses the capabilities of individuals with severe disabilities, supported work models, agency roles, transition procedures, and the effects of wages on benefits to facilitate smooth school-to-work transitions. Hoeffding et al. (26) adopt a more structured approach, offering SOs manualized training through eight 90-minute family intervention sessions and 12 h of individual caregiver coaching. This support is designed to help SOs apply the strategies they have learned in everyday life and at work, and it explicitly targets SOs in the intervention. Kelley and Lambert (21) describe that the type of support offered to families or caregivers varies across their included studies encompassing psychoeducation, medical information, skills training (e.g., coping, communication, problem-solving, patient management), counseling or group support, and pre-discharge education on prevention and coping strategies, awith SOs being directly involved in the different kinds of interventions. In contrast, other studies adopt different approaches. Brongers et al. (24) directly involve SOs in Family Group Conferences and inform them about intervention procedures, but do not provide systematic training. Snippen et al. (25) do not directly include SOs as a target group, but rather indirectly through training professionals. Likewise, McKenna and Power (23) and Chang et al. (20) primarily target professionals rather than providing direct guidance to SOs. Both studies address the role and involvement of SOs in the vocational rehabilitation and employment transition process, but neither offers an explicit intervention for this group.

## 4 Discussion

This scoping review shows that despite the importance of SOs for health, recovery processes as well as returning to and staying at work (9, 31), there are very few documented vocational rehabilitation interventions who explicitly and systematically include SOs in the return-to-work process. By “systematic,” we do not mean “standardized”. Given the diversity of SO constellations and relationships, individual approaches are needed. What we mean by “systematic” are interventions that provide guidelines for professionals, e.g., on the timing and methods of SO involvement, structured session content, and decision-making frameworks.

Similar gaps have been documented in related areas. White et al. (31), who examined return-to-work interventions, requiring explicit enhancement of social support or social integration, identified no eligible studies. The studies we were able to identify are very heterogenous regarding the targeted populations, theoretical foundations, and approaches for SO involvement.

However, it is possible that approaches do exist in rehabilitation practice but are not systematically researched and published. A lack of published research points to the importance of systematically investigating existing practices and making valuable knowledge of practitioners available to others. Otherwise, the evaluation of and (further) development of new as well as existing approaches is hindered. This is particularly relevant for school-to-work transitions, where the involvement of SOs is likely to extend beyond what is captured in peer-reviewed publications and is more likely to be found in grey literature. This gap may be further compounded by the exclusion of non-English resources, since knowledge of transition support structures specific to a given country is likely to be documented primarily in the national language(s) rather than in English. Therefore, future reviews could focus on school-to-work transitions and involve grey literature and multilingual sources to capture the full range of existing practice.

Further methodological factors may have contributed to the limited number of identified studies, such as the terminological heterogeneity surrounding the concept of significant others, with varying terms used inconsistently across the literature, which may have resulted in incomplete keyword coverage in our search strategy and the unintentional exclusion of relevant studies. The disciplinary fragmentation between occupational health and rehabilitation research presents an additional challenge, as relevant literature may be spread across distinct academic fields, further limiting the retrieval of studies across disciplinary boundaries.

Another explanation for the scarcity of intervention studies is that SOs may not be routinely included in practice, likely due to time and financial constraints. Workers themselves also raised concerns that limited consultation time could hinder active SO involvement (2).

Most of the interventions are not theory- or concept-guided. Well-established theoretical or conceptual approaches such as systemic coaching or therapy, social network theories, or empowerment concepts from Social Work are hardly made use of. Of the eight included studies, only three explicitly referenced a theoretical framework or concept. The theories mentioned included dyadic coping and illness perceptions (25), empowerment, strength and social network theory (24) and the vulnerability-stress model (21). The majority of interventions provided no theoretical rationale or supporting concepts for the involvement of SOs and do not specify which dimension of social support they aim to address or how SOs are expected to provide support for return-to-work-processes (e.g., emotionally, informationally, or instrumentally). This may prove a more practice-driven rather than theory-driven approach to the development of the intervention concepts. This conceptual imprecision hinders both systematic intervention development and the comparability of approaches.

The definition and selection of SOs is unclear. Explicit criteria for who counts as a SO and for who decides about whom to include are rarely mentioned. Usually, the focus is on the family, friends or other peers are not taken into account.

When included, SOs are primarily understood as (and included with the rationale of being) beneficial to the rehabilitants’ return to work. Potential risks such as overprotection, conflicts or dependencies are rarely addressed. Only one study mentions SOs as also a potential barrier to rehabilitants’ return to work. Health professionals and rehabilitants include SOs with the rationale that they improve communication, provide information, foster anchoring of rehabilitation and act as a resource for professionals, or are mandatory when rehabilitants are minors (19). Only Kelley & Lambert (21) recognize SOs as potential barriers, using their involvement to prevent dysfunctional family patterns. One study (26) also mentions the aim of reducing the caregiver burden, recognizing SOs as intervention recipients in their own right. Other studies do not address SOs as a primary target group, neglecting their role requirements and potential burdens.

These findings highlight several important implications for future research. First, there is a clear need for the development of theory and evidence-driven interventions for including SOs into vocational rehabilitation services. This could also start with systematically documenting and evaluating existing practices on how practitioners currently involve SOs. Third, newly developed interventions must include clear operational definitions of who qualifies as a SO, explicit inclusion criteria, and detailed role descriptions that specify what SOs are expected to do and how these activities support rehabilitation outcomes. Finally, research should adopt dyadic or systemic models that recognize SOs not only as instrumental supports but as individuals with their own needs, role requirements, and potential burdens.

### 4.1 Strengths and limitations

This scoping review is the first to systematically map vocational rehabilitation interventions explicitly including SOs in return-to-work processes and provides an understanding of current approaches and identifies critical gaps for future research and practice development. By following established scoping review methodology standards of Arksey and O’Malley (17) the review adopts a state of the art development process. Methodological transparency was ensured through protocol preregistration on the Open Science Framework and adherence to PRISMA-ScR reporting standards (18).

However, the review does have some limitations. The included studies are low in number and differ considerably. On the one hand, this variation is a strength, as it aligns with the intention of scoping reviews to map the scope of available evidence across diverse contexts, regardless of quality or methodology, but it also limits comparability of the intervention. As mentioned above, another key limitation is the potential for selection bias due to conceptual ambiguity and inconsistent terminology surrounding the involvement of SOs. This may have resulted in incomplete keyword coverage and the unintentional exclusion of relevant interventions. The issue is further complicated by the wide range of approaches to vocational rehabilitation, which include medical, psychological, and social components and are delivered in various settings by different professional disciplines. While relevant SO involvement may exist within specific rehabilitation subfields, it may not have been identified unless framed explicitly as vocational rehabilitation or return-to-work interventions. Possible publication biases may reflect a limited academic awareness of the topic and field. A lot of valuable information about everyday practice may be found in grey literature reports or guidelines, including printed, not available online material. Furthermore, the quality of reporting across studies was inconsistent. Many of the included studies did not explicitly describe key aspects of SO involvement, such as the selection criteria, the specific roles and how the involvement was structured. This required the interpretation of contextual information, which introduced potential bias and uncertainty.

Despite its limitations, this scoping review provides insight into the types of interventions involving SOs in occupational health that have been documented in peer-reviewed literature. Highlighting the scarcity of published interventions, as well as the lack of theoretical grounding and standardised approaches. However, as knowledge in vocational rehabilitation is often not translated into peer-reviewed research, it reflects only the scientific perspective and is unlikely to fully capture the range of existing practice. Given the significant importance of SO involvement in achieving successful rehabilitation outcomes, the disparity between practice and research highlights the urgent need for greater academic focus in this area.

## 5 Conclusion

Despite the widespread acceptance of the fact that rehabilitants’ SOs play an important role in the return-to or stay-at-work process, there is an astonishing lack of interventions that try to involve them in the rehabilitation process. Existing interventions are heterogeneous and rarely theory-driven. Future research should focus on the development and evaluation of structured, theory-informed approaches that integrate SOs in a targeted and needs-based manner, while also considering their potential burdens and roles beyond being a supportive resource.

## Data Availability

The original contributions presented in the study are included in the article/Supplementary Material, further inquiries can be directed to the corresponding author.
