# Supplementary material for: Including significant others in vocational rehabilitation: a scoping review
Source: Front Rehabil Sci. 2026 Jun 22;7:1833648. doi: 10.3389/fresc.2026.1833648 (PMC13333620; doi:10.3389/fresc.2026.1833648)
Supplement: Supplementary file 2 [file Table2.docx]

Supplementary Material

**Supplementary Table 2: Final Search Strategy**

**Pubmed May 14^th^ 2025**

| **Concept** | **Results** |
| --- | --- |
| "Family"[Mesh] OR "Caregivers"[Mesh] OR "Social Support"[MeSH] OR : Relative* [tiab] OR famil* [tiab] OR dependent* [tiab] OR caregiver*[tiab] OR kin[tiab] OR neighbo*[tiab] OR “significant other*”[tiab] OR “social support" [tiab] OR "informal carer*"[tiab] OR partner*[tiab] OR child* [tiab] OR “in-law*” [tiab] OR sibling* [tiab] OR spouse* [tiab] OR friend* [tiab] OR parent* [tiab] OR grandparent* [tiab] OR mother* [tiab] OR father* [tiab] OR grandmother* [tiab] OR grandfather* [tiab] OR aunt* [tiab] OR uncle* [tiab] OR cousin* [tiab] OR daughter* [tiab] OR son [tiab] OR offspring [tiab] OR kid [tiab] OR godmother* [tiab] OR godfather* [tiab] OR godparent* [tiab] |  |
| "Chronic Disease"[Mesh] OR "mental disorders"[MeSH Terms] OR Chronic* [tiab] OR “chronic condition*”[tiab] OR “chronic illness*”[tiab] OR “chronic symptom*”[tiab] OR “chronic disease*”[tiab] OR “long-term condition*” [tiab]OR “long-term symptom*” [tiab] OR “long-term illness*” [tiab] OR “long-term disease*” [tiab] OR “persistent condition*” [tiab]OR “persistent symptom*” [tiab] OR “persistent illness*” [tiab] OR “persistent disease*” [tiab]OR long-standing[tiab] OR disabilit* [tiab] |  |
| "Rehabilitation, Vocational"[Mesh] OR "Return to Work"[Mesh] OR “Vocational rehabilitation” [tiab] OR “occupational rehabilitation” [tiab] OR “return to work” [tiab] OR “stay at work” [tiab] |  |
| involv* [tiab] OR inclu* [tiab] OR engag* [tiab] OR integrat* [tiab] OR help* [tiab] OR participat* [tiab] address* [tiab] OR target*[tiab] |  |
| project [tiab] OR promot* [tiab] OR implement* [tiab] OR trial [tiab] OR evaluat* [tiab] OR intervention [tiab] OR program* [tiab] |  |
| #1 AND #2 AND #3 AND #4 AND #5 | **159** |

**Web of Science May 14^th^ 2025**

| **Concept** | **Results** |
| --- | --- |
| TS= (Relative* OR famil* OR dependent* OR caregiver*OR kin OR neighbo* OR “significant other*” OR “social support" OR "informal carer*" OR partner* OR child* OR “in-law*” OR sibling* OR spouse* OR friend* OR parent* OR grandparent* OR mother* OR father* OR grandmother* OR grandfather* OR aunt* OR uncle* OR cousin* OR daughter* OR son* OR offspring OR kid* OR godmother* OR godfather* OR godparent*) |  |
| TS= (Chronic* OR “chronic condition*”OR “chronic illness*” OR “chronic symptom*” OR “chronic disease*” OR “long-term condition*” OR “long-term symptom*” OR “long-term illness*” OR “long-term disease*” OR “persistent condition*” OR “persistent symptom*” OR “persistent illness*” OR “persistent disease*” OR long-standing OR disabilit* OR "mental disorders") |  |
| TS= (“Vocational rehabilitation” OR “occupational rehabilitation” OR “return to work” OR “stay at work”) |  |
| TS= (involv* OR inclu* OR engag* OR integrat* OR help* OR participat* address* OR target*) |  |
| TS= (project OR promot* OR implement* OR trial OR evaluat* OR intervention OR program*) |  |
| #1 AND #2 AND #3 AND #4 AND #5 | **555** |

**PsycInfo May 14^th^ 2025**

| **Concept** | **Results** |
| --- | --- |
| TI,AB(relative* OR famil* OR dependent* OR caregiver* OR kin OR neighbo* OR "significant other*" OR "social support" OR "informal carer*" OR partner* OR child* OR "in-law*" OR sibling* OR spouse* OR friend* OR parent* OR grandparent* OR mother* OR father* OR grandmother* OR grandfather* OR aunt* OR uncle* OR cousin* OR daughter* OR son* OR offspring OR kid* OR godmother* OR godfather* OR godparent*) OR SU.EXACT("Caregivers") OR SU.EXACT("Family") OR SU.EXACT("Social Support") OR SU.EXACT("Social Networks") |  |
| TI,AB(chronic* OR "chronic condition*" OR "chronic illness*" OR "chronic symptom*" OR "chronic disease*" OR "long-term condition*" OR "long-term symptom*" OR "long-term illness*" OR "long-term disease*" OR "persistent condition*" OR "persistent symptom*" OR "persistent illness*" OR "persistent disease*" OR "long-standing" OR disabilit* OR "mental disorders") OR SU.EXACT("Chronic Illness") OR SU.EXACT("Chronic Mental Illness") |  |
| TI,AB("vocational rehabilitation" OR "occupational rehabilitation" OR "return to work" OR "stay at work") OR SU.EXACT("Vocational Rehabilitation") OR SU.EXACT("Return to Work") |  |
| TI,AB(involv* OR inclu* OR engag* OR integrat* OR help* OR participat* OR address* OR target*) |  |
| TI,AB(project OR promotion OR implement* OR trial OR evaluat* OR intervention OR program*) |  |
| #1 AND #2 AND #3 AND #4 AND #5 | **415** |
