# Supplementary material for: Including significant others in vocational rehabilitation: a scoping review
Source: Front Rehabil Sci. 2026 Jun 22;7:1833648. doi: 10.3389/fresc.2026.1833648 (PMC13333620; doi:10.3389/fresc.2026.1833648)
Supplement: Supplementary file 3 [file Table3.docx]

**Supplementary Table 3:** Characteristics of included studies (long version)

| **Publication Details** | **Study Details** | **Outcome Measures** | **Results** | **Conclusion** |
| --- | --- | --- | --- | --- |
| **Reference:** Snippen et al. (2023)  **Title:** Training for occupational health physicians to involve significant others in the return-to-work process of workers with chronic diseases: a randomized controlled trial  **Country:** Netherlands | **Study design:** non-blinded randomized controlled trial (RCT), with an intervention and a wait-listed control group.  **Intervention Type:** E-learning training for occupational health physicians  **Aim oft he study:** evaluate the effectiveness of an e-learning training module for occupational health physicians on their knowledge, attitudes, and self-efficacy regarding involving significant others in the return-to-work process of workers with chronic diseases  **Sample:** occupational physicians and insurance physicians. (N= 87 | Knowledge 20-item multiple-choice test (max score 20)  Attitude 11 items on 6-point Likert scale (adapted from family involvement scale) Self-efficacy 15 items on 5-point Likert scale (adapted from clinical skills tool) Satisfaction with TOTIS Evaluation form (13 items) + open-ended feedback | **Quantitative Results:**OHPs' knowledge (p < 0.001, nр² = 0.202), attitudes (p = 0.003, np² = 0.098), and self-efficacy (p < 0.001, np² 2 = 0.237), with retention of all changes at 10-week follow-up. OHPs graded the e-learning module with a mean score of 7.9 out of 10 (SD = 1.11)  **Qualitative Results**: heightened their awareness of the role of significant others in the return-to-work process and  encouraged them to address this more often. | **Conclusion**: more awareness, new insights, greater motivation  improvement concerning knowledge about involving SO, attitude towards it, self-efficacy Practical tips and materials were valued. Criticism: navigation issues, tech problems,  lack of practice, feedback, and peer exchange.  **Perceived Methodological Limitations:** No direct measurement of actual behavior change or worker outcomes |
| **Reference:** Brongers et al. (2020)  **Title:** Feasibility of Family Group Conference to promote return-to-work of persons receiving work disability benefit  **Country:** Netherlands | **Study Design:** Mixed-methods feasibility pilot study (no control group) with measurements at baseline (T0), immediately post-intervention (T1), 3 months (T2), and 6 months (T3) follow-up  **Intervention Type:** Family Group Conference (FGC)  **Aim of the Study:** evaluate the feasibility of Family Group Conference  **Sample:** convenient sample of Labour experts, Clients, and Facilitators: 14 labour experts and 16 facilitators enrolled, 9 clients participated | 12-item Short Form Health Survey,Participation Ladder and Study-specific questionnaires developed for this feasibility study | **Quantitative Results:** Implementation: 78% of FGCs carried out as planned  Satisfaction: Mean rating ~7 (good) Outcomes: Slight improvement in mental health and participation level RTW plans: Mostly work-related; clients took lead, supported by their network After 6 months: 5 of 9 clients returned to paid or voluntary work  **Qualitative Results**: Positive perception of FGC:  Seen as empowering and motivating  Provided structure to explore RTW options  Increased awareness of social network support  Social support effects:  SOs felt engaged and valued  Enhanced communication within network  Barriers identified:  Timing too early for some  Health conditions limited progress  Limited participation due to fear or emotional difficulty | **Conclusion:** FGC facilitated RTW planning; positive experiences reported, perceived as helpful by majority  **Perceived Methodological Limitations:** Small sample size (9 participants), limiting generalizability, No control group; cannot attribute effects solely to intervention |
| **Reference:** Hoeffding et al., (2017)  **Title:** A manual-based vocational rehabilitation program  for patients with an acquired brain injury: study protocol  of a pragmatic randomized controlled trial (RCT)  **Country:** Denmark | **Study Design:** interventional, two-arm, six-month follow-up, cluster randomized controlled trial. Control group: conventional VR  **Intervention Type:** individually targeted vocational rehabilitation  **Aim oft the Study:** develop an individually targeted manual-based VR program and determine its efficacy for patients with ABI  **Sample:** 84 patients with acquired brain injury | hours at work/ study, proficiency on functional task, functional statues, health-related quality of life, anxiety, depression, fatigue, self-perceived burden to others, work ability, sociodemographic, intelligence, executive, cognitive, working memory quality of life, family interactions, problem-solving ability, caregiver burden | **Quantitative Results:** none yet  **Qualitative Results**: none yet | **Conclusion:** none yet  **Perceived Methodological Limitations:** none yet |
| **Reference:** Chang et al., (2024)  **Title:** Providers’ Perceptions of the Collaborative Challenges and Assistance Provided to Families in Pre-Employment Transition Services  **Country:** USA (Texas) | **Study Design:** qualitative, 24 in-person focus groups  **Intervention Type:** Service delivery context of pre-employment transition VR counseling  **Aim oft the Study:** explore providers' perspective on family involvement in pre-employment transition planning: challenges and assistance provided to families during transition to postsecondary education or work  **Sample:** 162 providers of Pre-employment transition services for youth with disabilities (51% transition educators, 36% VR counselors, 14% others); 84% female; >5 yrs transition experience: 52%; 24 focus groups, 6–12 per group | Qualtitqative themes: barriers and challenges to provide pre-ets, from schools, families, other support organisations, potential solutions; code frequencies; codebook; trustworthiness procedures (peer debriefing, coder training) | **Quantitative Results:** not applicable  **Qualitative Results**: 3 themes concerning barriers and facilitators:  (1) Need to Support Family Directly (knowledge/benefits, emotions, financial/resource needs);  (2) Need to Promote Family–Professional Communication (contact paths, language, divergent beliefs/expectations incl. rural/immigrant/non‑traditional families);  (3) Need to Promote Community Support (transportation, engaging religious/community orgs) | **Conclusion:** Providers emphasize tailored direct support to families,  culturally responsive communication,  and community resource development (esp. transport) to enhance Pre‑ETS family collaboration.  **Perceived Methodological Limitations:** Qualitative single‑state sample (Texas); nominated/convenience sampling may bias perspectives;  generalizability limited; focus groups emphasize barriers; recommend broader sampling and more facilitators-focused questions**.** |
| **Reference:** McKenna & Power (2000)  **Title:** Engaging the African American Family in the Rehabilitation Process: An Intervention Model for Rehabilitation Counselors  **Country:** USA | **Study Design:** not a study per se, sharing long-term experiences  **Intervention Type:** family intervention model  **Aim oft he Study:** developing an intervention model from long-term experience  **Sample:** not specified | not specified | **Quantitative Results:** not specified  **Qualitative Results**: not specified | **Conclusion:** importance of cultural awareness, adopting different roles  **Perceived Methodological Limitations:** not an structured study, sharing learnings from practical experience |
| **Reference:** McCarthy et al., 1985  **Title:** Transition from School to Work: Developing the Process for Individuals with Severe Disabilities  **Country:** USA | **Study Design:** step by step procedure to develop a transition process, descriptive case study  **Intervention Type:** Complex, multi-level intervention model for school-to-work transition  **Aim of the Study:** developing model for school-to-work transition process  **Sample:** study presents a conceptual framework plus one illustrative case (18-year-old male student) | No formal measurement instruments | **Quantitative Results:** No quantitative results – purely descriptive/case-based  **Qualitative Results**: Families perceived as crucial for motivation, treatment compliance, and adaptation. Positive reports from structured interventions (psychoeducation, training). Family attitudes strongly shaped patient experience | **Conclusion:** Employment for individuals with severe disabilities  is achievable  if transition planning is individualized, starts early, and  involves parents, schools, and community agencies  **Perceived Methodological Limitations:** no control, no sample, no measurement, limited generalizability |
| **Reference:** Snippen et al., 2022  **Title:** Workers’ views on involving significant others in occupational health care: a focus group study among workers with a chronic disease  **Country:** Netherlands | **Study Design:** Qualitative focus group study  **Intervention Type:** not applicable  **Aim of the Study:** To explore workers’ views on involving significant  others in occupational health care in the context of chronic disease  **Sample:** 21 workers with a chronic illness | semi-structured interviews about: perceptions, attitudes, perceived benefits and risks of involving significant others in occupational health care | **Quantitative Results:** Not applicable  **Qualitative Results**: Workers generally support involvement of SOs; perceived benefits include practical and emotional support, shared understanding, better communication with OHPs. Concerns include privacy, autonomy, possible burden on SOs. | **Conclusion:** Involving SOs can be valuable in occupational health care for workers with chronic disease,  but requires careful consideration of privacy, voluntariness, and clear role definition.  **Perceived Methodological Limitations:** Small sample, Dutch context only, qualitative design limits generalizability, compensation: €20 gift certificate for participating in the study |
| **Reference:** Kelley & Lambert, 1992  **Title:** Family Support in Rehabilitation: A Review of Research, 1980–1990  **Country:** USA | **Study Design:** Narrative review: includes observational, nonrandomized and randomized intervention studies, case studies, and surveys  **Intervention Type:** Review of diverse family support interventions across rehabilitation contexts (physical disability, mental illness, substance abuse, developmental disabilities) with different aims  **Aim of the Study:** to review and synthesize research on the role of family support in rehabilitation outcomes for persons with chronic illnesses and disabilities, published between 1980-1990.  **Sample:** Populations include patients with physical disabilities, long-term mental illness, substance abuse disorders, developmental disabilities, and their families/caregivers | Treatment compliance, psychosocial well-being, functional status, employment outcomes, family functioning, mortality, symptom control | **Quantitative Results:** Physical Disabilities:  Stroke caregivers → family support improved adherence; Sickle cell adolescents → less support = more hospital/narcotic use; Chronic disability → outcomes varied with actual vs. perceived support; Renal failure families → over-coordination worsened outcomes Mental Illness: 66% with long-term illness returned home; Family psychoeducation reduced hospitalizations (cost-effective) Substance Abuse: Family involvement improved retention, reduced relapse probability Developmental Disabilities: Intensive support reduced anxiety/depression; single parents more at risk of poor outcomes  **Qualitative Results**: Support functions identified: emotional concern, instrumental aid, communication, problem-solving, treatment compliance enhancement, stress buffering  Family impact: adherence, adjustment, well-being, employment success  Negative effects: overprotective/overly coordinated families hinder rehabilitation, enmeshment worsened disease control | **Conclusion:** Family support is critical for rehabilitation outcomes; Positive effects when families provide supportive involvement; risks when families are overprotective or dysfunctional. Emphasizes structured family involvement as best practice; systematic, controlled studies with standardized measures needed  **Perceived Methodological Limitations:** Inconsistent conceptualization of social support; heterogeneous definitions; unique instruments limited comparability |

**Table 2:** Intervention Characteristics (long version):

| **Reference** | **Theory and Target** | **Materials** | **Procedures** | **Delivery parameters** | **How and How much** |
| --- | --- | --- | --- | --- | --- |
| Snippen et al. (2023) | **Rationale/Theory:** OHPs rarely involve SOs in return-to-work processes due to lack of knowledge, skills, and self-efficacy. Rationale: to evaluate the effectiveness of an e-learning training module (TOTIS) in improving OHPs' knowledge, attitudes, and self-efficacy regarding involving SO  **Intervention Target:** Occupational health physicians | (1) a reference book,  (2) validated questionnaires,  (3) a conversation leaflet, and  (4) ten disease-specific leaflets | (1) when and how to address the role of significant others;  (2) coping and re- integration;  (3) the role of dyadic coping;  (4) the role of illness perceptions; and  (5) summary of key messages and best-practice recommendations. Content within each part was focused on deliv- ering essential knowledge and translating that knowledge into practical skills (i.e., the “know” and “do” for best-practice in involving significant others). | **Timing:** when to involve significant others in a worker's return-to-work process is left to the trained OHP's clinical judgment based on individual assessment of the worker's needs and circumstances  **Who provided:** provided by researchers at the University Medical Center Groningen  **Where:** online module,  accessed by participants  at their own preferred timing,  location, and speed | **How:** was delivered individually via the internet through an e-learning module  **How Much:** Not specified; participants completed it at their own pace within the 4 weeks. |
| Brongers et al. (2020) | **Rationale/Theory:** Individuals on work disability benefits face persistent barriers to labor market re‑entry and often experience fragmented, professional‑driven support that underutilizes existing social networks. Rationale: to assess the feasibility and acceptability of involving family /social networks through Family Group Conference promoting return to work  **Intervention Target:** participants were aged 17–65 years, receiving work disability  benefit, had (partial) capacity to reintegrate into paid work  according to their Labour Expert, but were not self-reliant in find-  ing work | unclear | Beginning: professionals (-Labour Expert) provide clear, brief information on work ability, benefits, and options; questions are addressed.  Private family time: Facilitator and professionals withdraw; client and network discuss needs, resources, and agree on a concrete return-to-work plan (actions, timelines, responsible persons).  Plan confirmation: Facilitator returns to record the plan, responsibilities, and follow-up arrangements; copies shared with all parties.  Follow-up: Labour Expert and client monitor execution; adjustments made as needed at 3–6 months. | **Timing:** was not linked to the onset of the condition  **Who provided:** The  Family Group Conferences were carried out by the clients, their  families, the Labour Experts from the Social Security Institute  involved in the vocational rehabilitation of these clients, and the  Family Group Conference facilitator.  **Where:** Community setting, typically in participants’ home or a chosen venue | **How:** Face-to-face;follow-up interviews by telephone  **How Much:** 2 months from intake to follow-up: Intake, preparation, FGC session, RTW plan creation, follow-up 1 FGC session per client; plus 2 follow-up contacts |
| Hoeffding et al. (2017) | **Rationale/ Theory:** reduce caregiver burden, support the rehabilitant & the profesional team, make retention/consolidation of work more likely. Bi-directional relationship between patient and family wellbeing  **Intervention Target:** patients with acquired brain injury and their family caregivers | unclear | family intervention program, individual caregiver coaching | **Timing:** 3-24 months after acquiring the brain injury  **Who provided:** rehabilitation team (not specified which profession)  **Where:** municipality/ workplace/ home | **How:** both individually and in group, face to face  **How Much:**8 90-minute family intervention program sessions, 12 hours individual caregiver coaching |
| Chang et al. (2024) | **Rationale/Theory:** Family involvement in transition planning, which is legally required and linked to improved outcomes such as competitive employment, makes it important to examine providers’ perspectives on family-related barriers and facilitators in delivering Pre-ETS to youth with disabilities  **Intervention Target:** youth with disabilities before transitioning into work or post-secondary education and their families | not applicable | counselling | **Timing:** transition period from high school to postsecondary  education or work  **Who provided:** VR counselors, transition educators  **Where:** unclear, probably schools, homes or VR institutions | **How:** unclear  **How Much:** unclear |
| McKenna & Power (2000) | **Rationale/Theory:** family involvement can be a resource or potential barrier, african american families might have specific reservations concerning VR etc., function differently  **Intervention Target:** black persons with disabilities and their families | unclear | counselling, assessment, provision of information | **Timing:** not specified  **Who provided:** rehabilitation counselors  **Where:** unclear, probably schools, homes or VR institutions | **How:** unclear  **How Much:** unclear |
| McCarthy et al. (1985) | **Rationale/Theory:** Successful school-to-work transition requires a coordinated, community-based approach, individualized transition plans (ITPs), real-work training and collaboration among schools, families, and agencies ensures continuity of support, Long-term follow-up  **Intervention Target:** youth with disabilities/ mental retardation who are transitioning from school programs to employment. | unclear | assessment, counseling, education | **Timing:** best start = age 16 during secondary school  **Who provided:** special education Teachers, Rehabilitation counselors/case managers,Disability support services, Ancillary staff (e.g., speech, occupational therapy),  Parents / guardians, Employers  **Where:** schools/ job site, agencies, and family settings | **How:** both individually and in group, face to face  **How Much:** -Typically from age 16 until school completion (up to age 22); case study: ~3 year - ITP updated annually (or more often); team meetings regularly; job training ≥4 hrs/day at job site  -Planned overall length: multi-year process (2-year pilot trial, extendable to entire school-to-work transition) |
| Snippen et al., 2022 | **Rationale/Theory:** Workers with chronic diseases face challenges at work; SOs may provide emotional and practical support. Rationale: to assess acceptability and perceived value of SO involvement in occupational health care.  **Intervention Target:** Workers with chronic illness and their significant others (partners, family, close friends). | not applicable | guided integration of SO into occupational health care systems | **Timing:** not specified: Potentially at various stages of occupational health care: diagnosis, return-to-work planning, ongoing work support.  **Who provided:** occupational health professionals  **Where:** occupational health care | **How:** unclear  **How Much:** unclear |
| Kelley & Lambert, 1990/1992 | **Rationale/Theory:** Lacks a unified theoretical framework; review shows importance of family support but no single theory  **Intervention Target**: Persons with chronic illness/disability (physical, mental, substance abuse, developmental)  and their significant family members | Informational booklets,  educational materials for families, pre-discharge guides, psychoeducational resources, stress management manuals, structured questionnaires | Physical: pre-discharge education for caregivers; Mental illness: Inpatient Family Intervention (IFI), psychoeducation, communication/problem-solving, medication compliance; Substance abuse: counseling, social support; Developmental: case management, coping skills, behavioral parent training | **Timing:** Pre-discharge from hospital/inpatient settings (stroke, SCI). During acute rehabilitation (family adaptation critical). Post-hospital/community transition (psychiatric patients returning home). Throughout rehab for chronic conditions. Early intervention for SCI counseling. General: flexible, across the rehabilitation continuum.  **Who provided:** Rehabilitation professionals (social workers, physicians, therapists, psychologists, psychiatrists, nurses, case managers, sometimes volunteers);  many trained in family systems/psych rehab/counseling  **Where:** Hospitals, inpatient rehab units, outpatient clinics, patient homes, community centers | **How:** Primarily face-to-face,  individual and group formats  **How Much:** from single session to months; Essential elements: multiple psychoeducational sessions, group meetings, skill-training; Frequency: from once at discharge to weekly; Length: one-time to continuous through rehab and readjustment |
